# Providing recovery support to wounded, injured, and sick UK military personnel throughout the COVID-19 pandemic

**DOI:** 10.1080/08995605.2022.2126655

**Published:** 2022-10-04

**Authors:** Christopher W. P. Kay, Rebecca J. Sutton, Gemma L. Margerison, Jim McKenna

**Affiliations:** Carnegie School of Sport, Leeds Beckett University, Leeds, UK

**Keywords:** COVID-19 pandemic, recovery, adaptive sport, mental health, health coaching, psychological well-being, behavior change, military recovery, psychosocial development, physical activity, wounded, injured & sick military personnel

## Abstract

Health precautions implemented by the United Kingdom (UK) government to limit the spread of the Coronavirus Disease 2019 (COVID-19) led to the closure of many well-being support services in 2020. This created a need to re-think how impactful recovery support courses can be provided. One such service was that of the five-day Multi Activity Course (MAC) which was redesigned in accordance with national health guidelines to allow continued access for Wounded, Injured and Sick (WIS) military personnel to the service; the positive impacts of which are well established. This study investigated the influence of the newly developed Reduced numbers MAC (R-MAC) on the WIS participants lives during and for 12 months after attending. The R-MAC led to comparable impacts for participants well-being, at a time in which people’s mental well-being was often being adversely affected. The positive mental well-being of the 261 participants improved by 33% throughout the course and remained 14% higher for the 37 participants who provided data six months after attending. Key facets of the experience that were most impactful for the participants were (i) shared experience with other veterans, (ii) discussing issues in a safe environment while receiving support from the staff and (iii) developing knowledge around self-help/personal development. Adapting to the challenging circumstances and developing the R-MAC mitigated against the already adverse impact of the COVID-19 pandemic for the WIS participants.

**What is the public significance of this article?—**The lessons learned from the recovery support provided at The Battle Back Center throughout the COVID-19 pandemic have far reaching implications beyond the military context. Adapting the courses to exceed COVID-19 restrictions meant that hundreds of recovering military personnel were still able to access this impactful support program. These positive program outcomes, shown both quantitatively and qualitatively, and over prolonged periods, show that COVID-19 protections were no barrier to positive and prolonged personal development. Indeed, the positive findings, especially the unexpected positivity emerging from working in smaller groups due to social distancing, catalyzed by regular monitoring of COVID-19 protections, ensured the R-MAC had valuable influences on these participants’ lives.

## Introduction

### COVID-19 public health restrictions

Pandemic status was declared on March 11, 2020 by the World Health Organization regarding illnesses caused by COVID-19. Worldwide implementation of strict social distancing controls aimed to limit transmission and avoid overwhelming health services (Douglas et al., 2020; Flint et al., 2020; Ghebreyesus, 2020). In the UK, additional guidance was issued by General Practitioners and health care professionals instructing “high risk” populations to shield. Shielding meant not leaving their home at all and minimizing all non-essential contact with other members of their household. People living with a disability or susceptibility to illness were recommended to shield, this affected many WIS military personnel (Douglas et al., 2020; Flint et al., 2020; Murphy et al., 2020). Contracting COVID-19 was associated with declines in general mental health and well-being as was adhering to the control measures in shielding groups (Durcan et al., 2020; Flint et al., 2020).

The enforced social isolation was described as a “*pervasive lack of social contact or communication*,” guaranteed to increase mortality, with even greater effects for those socioeconomically disadvantaged or of ill-health (Douglas et al., 2020, p. 2; Durcan et al., 2020; Flint et al., 2020; Holt-Lunstad et al., 2015). The uncertainty and unpredictability of the pandemic alone caused many to feel fearful and insecure, exacerbating psychological distress and delay in recovery from health care issues (Lu & Lin, 2021; Xiong et al., 2020). COVID-19 related restrictions also included significant changes in healthcare delivery with closure of non-clinical and non-essential services (Leite et al., 2021; Mantena & Keshavjee, 2021). It is also likely that the requirement for individuals to intentionally isolate themselves will have resulted in widespread social deconditioning (De Biase et al., 2020). Since social interaction restrictions have eased in the UK, retrospective research will highlight how the mental health consequences of such situations can be mitigated for at-risk communities in the future (Han et al., 2020; Holmes et al., 2020; Reger & Rothbaum, 2020).

### Impact of COVID-19 on recovering military personnel

The introduction of nationwide travel and social restrictions marked the beginning of significant disruptions to citizens lives in the UK. In line with health guidance, many military Personnel Recovery Centers (PRCs) closed, including the Royal British Legion (TRBL) funded Battle Back Center. While some centers responded by developing online remote support for beneficiaries (a wider phenomenon known as “Zoom boom”), many providers of services that could not moderate lockdown effects were closed, leaving beneficiaries and providers with uncertain futures (Help For Heros, 2021; Ruiz et al., 2020).

Personnel serving in the UK military, like other frontline staff and key workers, can experience heightened levels of stress, fear, and anxiety from crises such as the COVID-19 pandemic (Gary et al., 2020; Guo et al., 2020). Their mental well-being is particularly at risk in instances of quarantine, grief, loss, and exposure to the virus (Dubey et al., 2020). Prior to the pandemic, military personnel were known to seek support from doctors, medical officers, social workers, counselors, and their chain of command (Stevelink et al., 2019). Support from sub-clinical services was already increasingly being accessed pre-pandemic, including support from friends, family, chaplains, and charities (Iversen et al., 2010). The closure of sub-clinical support services for recovering personnel serving in the UK military brought with it the distinct likelihood of exacerbating their mental and physical health care needs.

### Previous impact of battle back MACs

One extensively accessed recovery support service for UK military personnel that has been available since 2010 is the MAC that is provided at TRBL’s Battle Back Center. By March 2020, MACs had already been attended by over 4,500 participants, with 18 further MACs planned for 2020. MACs are five-day residential support courses, based on adaptive sport and adventure training, designed to provide a context for personal growth, development, and recovery support. This is coupled with health coaching with the same staff throughout the five days. The bespoke MACs were developed in 2010 to provide additional support to recovering serving UK military personnel. Since 2010, studies have shown that these recovery courses recurrently facilitated positive and widespread changes in how participants described themselves and their lives, suggesting they engender increased adaptivity (Carless et al., 2013; Kaiseler et al., 2019; Kay & McKenna, 2022; Peacock et al., 2018, 2019; Sutton et al., 2021).

Narrative research with MAC participants has given in-depth clues into how the course facilitated increased cognitive flexibility, resilience, and overall behavioral adaptability (Carless, 2014; Carless et al., 2013, 2014). For some, the MAC led to a transformation in personal narratives (Frank, 2013), progressing from a failing monological narrative, through a Chaos narrative, toward a highly distinctive and functional, Quest narrative. This was exemplified in participants accounts of their experiences throughout a MAC. For example, prior to the MAC, participants have expressed thoughts such as “well that’s it, I’m disabled” and “I really don’t now have a clue what I can actually do now.” A shift in language of participants from comments such as “I was just nowhere” to the MAC having “opened some doorways in my head” point toward an altering personal narrative. Evidence in the accounts links the immersion in the physical activities and psychoeducation to the narrative development from Chaos to Quest for the participants. As part of this emerging understanding, participants increasingly linked their development to a greater sense of purpose, which supported persistence and commitment, including during setbacks, in different activities. This development was often contextualized by supportive MAC-based inter-personal relationships.

Based on narrative theory, this MAC-inspired transformation has had positive implications for the health and well-being of the participants (Carless, 2014; Carless et al., 2013). Personal accounts have consistently linked the benefits, meaning, and value of adventurous training and sport by illuminating individual experiences since injury or trauma, to their experiences while on the MAC, and how these have interacted to shape subsequent psychological well-being (Carless et al., 2014). Beyond subjective indicators of success, quantified measures of mental well-being have also detected improvements both within the five-day course (Peacock et al., 2018) and for many, sustained significant improvements over the 12 months following attendance (Kay & McKenna, 2022). Recent qualitative longitudinal research has evidenced the success of the MAC focus on “transfer effects” beyond the immediate experience of the course; many participants report sustaining positive behavioral changes made in the six (Kaiseler et al., 2019), or even the 12 months, following MAC attendance (Sutton et al., 2021).

The substantive evidence of the immediate and mid-term impact of the MAC led to it becoming a mandated part of the recovery pathway for Army and Royal Air Force (RAF) WIS personnel in 2012 and 2017 respectively. It is also strongly encouraged for Royal Navy personnel in recovery. Yet, due to UK health guidance linked to COVID-19, this recovery support service was suspended in March 2020. Given the record of accomplishment of positive impacts on the lives of many beneficiaries, there remained an ardent desire to develop an altered version of the MAC to continue to deliver what has been described as a “cornerstone activity for WIS service personnel.”^1^ (Ministry of Defence, 2020).

### Developing the R-MAC

With closure of the center, in line with Government health guidance regarding COVID-19, stakeholders immediately began to redevelop the MAC into the R-MAC. This rapidity was essential as the Battle Back Center is a functioning work environment for recovering service personnel, and as military personnel, their health and well-being needs would continue; when attending a recovery course, the main duty of military personnel is to purposively participate in the support.

When the MAC was established, it was intentionally not delivered at a closed military site, but instead, at the National Sports Center, to allow for social interaction with other members of the public. Off-site activities were chosen specifically to allow opportunities for interaction and engagement in the community during the five-day course; something that many participants reported avoiding since being down-graded due to their injury or illness. This included stopping at a café while traveling to and from physical activity venues, as well as a cinema trip. These are seemingly simple activities that were very meaningful and often challenging. They allowed the participants to re-engage in public environments with the support of the coaching staff. These community and public interactions were not possible during the R-MACs.

It was regularly reported that spending time with other people on the MACs who are also recovering was very valuable to the attendees. Being able to meet 23 other recovering personnel provided opportunities for shared experiences both during the course as well as being able to reflect on past experiences with others in similar situations. The first five R-MACs only had eight participants because of public health restrictions. The number of fellow WIS personnel the attendees could meet and potential benefit from interacting with was lower than they would have experienced on a pre-COVID-19 MAC that had 24 participants.

The public health measures that would need to be put in place to be able to deliver an R-MAC also brought drastic change to the previous MAC format. Namely, mask wearing, daily COVID-19 lateral flow testing, regular hand sanitization, daily temperature checks and social distancing at all times. All these elements posed a risk of impacting on the achievable well-being benefits a newly developed R-MAC could facilitate compared to the known success of the previous MACs.

Importantly, the development of the R-MAC prioritized restricting social interaction by mandating social distancing. As a result, R-MACs were initially limited to eight participants per course, working in two groups of four with two staff members. This ratio of 2:4 (staff:participants) aligned with the “rule of six” that had been advised for persons interacting indoors in a work environment. Virtual on-boarding to the course managed by Ministry of Defense (MoD) staff were delivered to ease pre-course apprehensions, social deconditioning effects from social-isolation and manage course expectations. All activities were delivered on-site to eliminate shared travel during the course that was previously utilized to travel to public sports facilities. A reduced-contact research approach was implemented, with each participant using a dedicated iPad to complete research surveys throughout the duration of the course, scanning QR codes to load the relevant survey, instead of research staff handling all participants iPads limiting the chance of contamination of physical objects and any potential infection transmission.

The adaptive sports and adventurous activities were changed to allow increased outdoor activity and adhere to and, where possible, exceed the government and individual sport’s National Governing Body (NGB) health guidelines. Activities included biking, archery, slack lining, orienteering, walking and golf. Regular sanitization intervals during activities took place, with cleaning and isolation of equipment for 72 hours between each use where necessary. Wheelchair basketball and seated volleyball were reintroduced once the sport’s NGBs guidance permitted.

While developing and delivering a COVID-19 health guideline compliant course was imperative, it was equally important to secure and to evidence whether R-MACs stimulate similar or greater health and well-being impacts to the previous MACs. Through a process of continuous discovery, focused on meeting the needs of participants, and to meet changing COVID-19 restrictions, the same key principles used to develop the MAC were used to refine the R-MAC. These included safety, trustworthiness and transparency, support and connection, collaboration and mutuality, empowerment, voice and choice, social justice, resilience, growth, and change (Elliott et al., 2005; Griffin, 2020).

Once the initial R-MAC design was finalized, approval from all major stakeholders was needed before delivery could commence. Relevant approvals were obtained from TRBL, Leeds Beckett University and SERCO Leisure, who manage the site on behalf of Sport England. MoD Regional Command gave their approval based on the recommendations of the Commanding Officer of the Personnel Recovery Units, Wales and West.

The first R-MAC was delivered in August 2020; four months after lockdown was initiated. As health guidance lightened, participant numbers were raised progressively from eight to 12, then to 16. As numbers increased, sporting and adventurous activities were re-introduced in line with updated health guidance that allowed, for example, group travel to public sports facilitates.

### Aims of the study

Once the R-MACs began to be delivered, it was vital to investigate the impact they had on the participants’ lives. They key aim of the study was to understand whether attending an R-MAC had positive influence on participant’s recovery and mental well-being. As well as pre-post outcome measures, this study investigated the active beneficial processes of the R-MACs that participants felt had the greatest impact on them. It was also critical to study if the participants felt the design of the R-MAC and the precautions put in place to safeguard against the transmission of COVID-19 were satisfactory. With these aspects considered, the study then aimed to reflect on the decisions made and lessons learned in preparation for future circumstances that can be managed with greater readiness and resilience to maintain vital support for recovering military personnel.

## Methods

### Participants

All participants were serving members of the UK military at the time of attending the R-MAC and were in recovery due to physical or mental illness or injury. Pre-course data sets were provided by 294 participants between August 17, 2020 and December 3, 2021; 81% were male and 19% were female, ranging in age from 19 to 63 years (mean = 36.35). The military distribution of the participants was 61.6% Army, 26.4% RAF, and 12% Royal Navy. The lowest ranks were of Army privates, RAF aircraftsmen and Navy able seamen. The highest-ranking participant was an Army Colonel.

Of the 282 participants who reported their WIS status, 80.85% were sick (mental health problem/physical illness), 31.91% injured (non-battle casualty) and 4.61% wounded (battle casualty). Participants could report multiple indicators, which explains why this total exceeds 100%; 331 responses were collected from 291 participants.

A total of 294 participants provided pre-course data and 305 sets of post-course data were collected. Both sets of data were screened to remove duplicate uploads, data sets that were only provided by a participant at one time point and incomplete data. This provided 261 complete paired pre-post data sets for analysis.

By December 31, 2021, the 165 participants who had attended an R-MAC six months ago (i.e., before July 1, 2021) had been emailed and invited to participate in follow-up research. Of the 165, 38 responses were received, giving a response rate of 23%. During data screening to pair these responses to their arrival data, one was removed due to duplication.

### Participant consent and data collection

To address voluntary consent, a participant information sheet and consent form were provided to all participants more than 24 hours prior to attending the Battle Back Center. On arrival, a verbal reminder and invitation to participate was provided by a research assistant; following which informed written consent was obtained. Participants confirmed their on-going consent prior to completion of follow-up surveys.

Immediately following the research reminder and completing informed consent, participants completed the Warwick-Edinburgh Mental Well-being Scale (WEMWBS; Tennant et al., 2007) to provide a baseline measure of mental well-being. Participants provided data at the end of the R-MAC for a pre-post comparison, and again, via online-survey, over the next year. This process mirrors how the WEMWBS data was captured during the MACs that had been running until the forced closure of the center in March 2020 and subsequent development of the R-MACs. Beyond the WEMWBS, participants were also asked to reflect upon their Battle Back experiences and the role those experiences have played within their recovery to-date, through a series of open-ended questions, at four follow-up time points over the next 12-months: two weeks, three months, six months and 12 months later.

The WEMWBS is an extensively validated psychometric scale proven appropriate for measuring positive mental well-being within serving military populations in the UK and US (Everill, Bennett & Burnell, 2020; Peacock et al., 2019; Roberts et al., 2019). It provides high internal consistency, with a Cronbach alpha level of 0.91 for general UK populations. It has also been demonstrated to provide high reliability using intra-class correlation coefficients (0.89; Tennant et al., 2007). The positively worded 14-item scale includes well-being components of positive affect, relations with others, and functioning from both hedonic and eudaemonic perspectives. Quantitative responses are measured on a five-point Likert-type scale, from one to five representing “none of the time” to “all of the time” respectively and are summed to indicate a low or high positive mental well-being.

### Data screening and analysis

Unique four-digit ID numbers were used to pair pre-post course WEMWBS data sets. Both pre-post course data comparisons and pre-course to follow-up comparative data sets were screened to create paired data sets by removing duplicate uploads, data sets that were only provided by a participant at one time point and incomplete data. Paired data sets were analyzed using a paired-sample t-test using IBM SPSS Statistics Version 27 software. Comparative analysis on unpaired data sets from MACs were analyzed using an independent t-test.

Thematic analysis was conducted on the written answers to open-ended questions (see Appendix 1) regarding participants reflections of the course aspect which had the greatest impact on them. This was done using Braun and Clarke’s six step thematic analysis method: (i) data familiarization, (ii) initial coding, (iii) generating themes, (iv) validity and reliability of themes, (v) defining and naming themes, and (vi) interpretation and reporting findings (Braun & Clarke, 2006). The data was categorized into codes, which were then grouped into coding sets based on key themes. These themes were identified through blinded analysis; three analysts independently conducted the first four steps of thematic analysis while steps five and six were undertaken collectively. From this data-driven inductive analysis, themes began to emerge allowing for coding interpretation of the data beyond the constrictions of pre-existing code frames.

### Ethical approval

Ethical approval was awarded by Leeds Beckett University and The Ministry of Defense Research Ethics Committee (MoDREC) (Protocol number: 562 MoDREC 14). MoDREC is an independent body comprising of MoD and non-MoD members, expert and lay. The committee operates according to guidelines set out by the UK’s Health Research Authority. Prior to final review and approval by MoDREC, scientific and technical rigor is assured through assessment by the appropriate Scientific Advisory Committee.

## Results

### Significant improvements in participants mental well-being pre-post R-MAC

**Figure 1.**
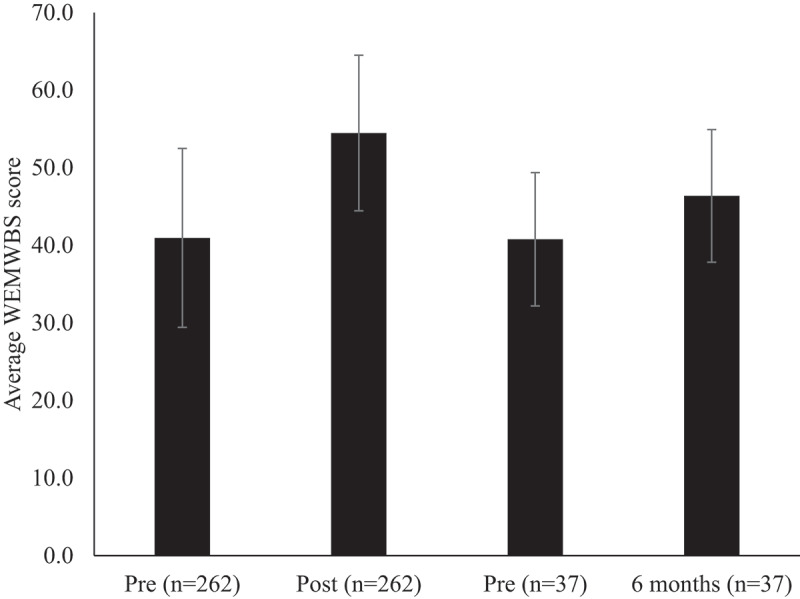
Paired WEMWBS scores of R-MAC participants pre-post course and paired data of those who completed the WEMWBS six months after.

The average pre-course mental well-being score of the 261 R-MAC participants was 40.95 (± 11.54) (*M* ± *SD*). This average was comparable to and not statistically significantly different to the average score of the last 261 participants that attended a MAC before the closure of the Battle Back Center in March 2020 and subsequent development of the R-MAC; 39.54 (± 11.98) *t*(520) = 1.435, *p* = .152 (independent samples t-test.). By the end of the five-day R-MAC the average increased significantly by 33%, *t*(260) = 14.69, *p* < .001 (paired samples t-test) as shown in Figure 1. This increase was comparable to the last 261 participants who attended a MAC before the closure of the center due to the pandemic, which was 36%.

### Long-term impact of the R-MAC

Participants were also invited to complete the WEMWBS survey again, six-months later, generating 37 paired data sets. This allowed for comparative analysis of the participants pre-course scores to their scores six-months later. The average pre-course well-being scores of these 37 participants was 40.78, comparable to all 261 participants' pre-course average of 40.95. The average well-being scores of the 37 participants who provided data at six months remained 14% higher at 46.38 (± 8.55) (see, Figure 1). This sustained improvement was statistically significant, *t*(36) = 4.16, *p* < .001 (paired samples t-test).

While completing the research surveys, participants were all provided an opportunity to write any additional comments they had about their experiences on the R-MAC. See some examples below;

“This course saves lives, you may never be able to quantify how many, but it really helps and makes a difference.” - three months

“This course has changed my life. Without it I may not have been here in a few months’ time.” - end of the course

“Truly believe without Battle Back I may not be here today.” - two weeks

“Since attending the course I have stopped … suicidal thoughts” - 12 months

“This course I feel is probably the most important of all courses I have been able to attend to date.” - three months

“This has been life changing for me and although I am not 100% yet, I do feel that I have the MAC to remember and fall back on if I have difficult times.” - six months

“This course was a game changer for me. I feel more motivated to carry on and be better” end of the course

“It was the missing piece in the puzzle that I needed to help me reset and take that step forward into getting back out there” - six months

Six months after attending an R-MAC, participants were asked to complete the following sentence “*In my recovery so far, the R-MAC was …* ” by choosing one of five options: *a waste of time, not helpful, OK, helpful or very helpful*. From 37 responses 49% chose *very helpful*, 38% selected *helpful*, 10% chose *OK* and 3% selected *a waste of time* or *not helpful*. Participants were also asked about the personal impact of the R-MAC since it ended. Respondents could choose from the five options of *negative impact, mostly negative, no impact, mostly positive*, and *positive impact*. From 37 responses 48% chose *mostly positive*, 38% selected *positive*, 10% chose no impact, one individual chose *mostly negative* and none selected *negative impact*. The sustained positive responses from participants regarding the impact of the R-MAC on their recovery are comparable to those of the MAC (Kaiseler et al., 2019; Sutton et al., 2021).

### The active beneficial processes of the R-MAC

Besides changes in participant mental well-being, the follow-up surveys also sought to understand the active beneficial processes of the R-MAC that may have facilitated the changes in participants well-being. Three months after, individuals were asked “What part of the Reduced-Multi-Activity Course had the greatest impact on you?” Three dominant themes emerged through analysis of the qualitative responses; (i) shared experience with other veterans, (ii) discussing issues in a safe environment while receiving support from the staff and (iii) developing knowledge around self-help/personal development. The themes and codes identified through the blinded analysis are presented in Table 1, along with quotes which represent each theme.

**Table 1.** Qualitative analysis of open-ended responses from R-MAC participants who attended three months ago. They were asked, “What part of the reduced-multi-activity course had the greatest impact on you?”

Themes	Shared experience with other veterans (n = 25)	Discussing issues in a safe environment while receiving support from the staff (n = 15)	Developing knowledge around self-help/personal development (n = 14)	Physical activities (n = 6)	Environment (n = 2)	Could not choose (n = 1)	The activities facilitating meaningful discussions or thoughts around personal development (n = 4)	Respite (n = 1)
Codes	Social interaction, community, relatedness, like-minded, sense of belonging, companionship, friendship	1:1 support, coaching, support, approachable, safe environment	Gaining confidence, routine, positive mindset/thinking, coping skills, control, acceptance, pride, reflection, ability focused, emotional processing	Physical activity, teamwork, trying new things	Environment, non-judgmental, atmosphere	Everything	Activities, future planning, perspective, prioritization, talking, processing, well-being, speaking freely, opening up	Respite
Example quotes	“Bonding with others who are in the same situation as me” “Being around a group of people who all had their own issues felt supportive” “Meeting others in the same place as me and knowing I’m not on my own”	“Having an open place to speak freely and release some of the things that I was scared to talk about prior to the course” “The discussions surrounding where we are in our emotional recovery” “Talking about my challenges and taking ideas on how to overcome without being judged”	“Letting go of the past to get to the future” “Getting a chance to refocus and making steps towards a more positive outlook” “Learning how to be more positive about myself and my abilities”	“Introduced me to cycling which I haven’t really done since I was a teenager. I now regularly cycle 100 miles per week” “Doing the activities in general”	“The environment and relaxed atmosphere of the course” “Just being in the battle back environment”	“All of it”	“The golf coaching has changed my life” “ the introduction of well-being techniques was well mixed in with the sport side of things” “The morning walk, useful framing point to the day”	“Getting a break from work”

The first theme was socially-based and related to meeting, or being around, like-minded people. Most participants explained that a powerful impact of the R-MAC was to create situations and activities the participants could share and discuss. Through being encouraged to socialize, within distancing guidelines, the R-MAC initiated new friendships for some participants. This was often contrasted to “normal life” where this did not happen. This social aspect also helped some participants to rekindle the lost feeling of being “part of a team again.”

The second major theme was that of feeling supported and able to open up. Participants noted that coaches shared experiences either of military service or through their own personal struggles and recovery journeys. In turn, coaches reportedly focused on taking a more positive outlook on their lives and futures; this was often linked to doing something new and refreshing, and/or to re-engage with an activity they had undertaken prior to them becoming WIS. The R-MAC provided a safe space to talk. This safety underpinned several other themes, including being around like-minded people, being able to connect and to challenge themselves by establishing and then undertaking activities at their level of ability. Many reported that a specific activity or event created a context for a meaningful and deeper discussion either with other participants or coaching staff.

The third dominant theme related to learning and developing knowledge around self-help and personal development. Participants valued learning new strategies, theories, and techniques to use in their recovery, for example, being able to plan or to think positively about the future was not only valued but also directly linked to discussions and connections with coaching staff. Experiencing an initial boost or a growth in one’s own belief in themselves was a further factor in program impact.

### Participant satisfaction regarding COVID-19 safety

On the final day of the R-MAC, participants were asked how well they felt social distancing was managed in various aspects of the course, see, Table 2. It shows the participants opinions of how well managed this was in four distinct aspects of the course.

**Table 2.** Participant perceptions of how social distancing was managed during the R-MAC.

	Very well managed	Well managed	Neutral	Badly managed	Very badly managed	Total
During sports activities	71.38%	23.91%	4.04%	0.00%	0.67%	297
During classroom-based talks and presentations	75.43%	23.21%	0.68%	0.00%	0.68%	293
Completing the research surveys	75.09%	23.21%	1.02%	0.00%	0.68%	293
At mealtimes	77.13%	19.11%	2.73%	0.34%	0.68%	293

All participants were also asked whether they found the COVID-19 precautions put in place satisfactory. Of 297 respondents, 293 indicated *Yes*, 3 *No* and 1 *I am not sure.*

## Discussion

The newly developed R-MAC positively influenced the mental well-being of the WIS participants. This positive influence was sustained throughout the six-month follow-up study. Importantly, the R-MAC was delivered in a COVID-19 compliant way while also delivering similar impacts to those associated with its precursor, the MAC. With careful planning that adhered to the mandated COVID-19 regulations, the R-MAC successfully and intentionally sequenced participants through feeling psychologically safe, to experiencing positive emotions and seeing others share similar experiences, to re-integrating at a social level. This addressed the urgent need for research to investigate how mental health consequences for at-risk adults such as recovering service personnel can be mitigated during such pandemic conditions (Holmes et al., 2020).

### Interpretation of WEMWBS scores

Average baseline WEMWBS scores for the R-MAC participants during the pandemic were comparable to those drawn from pre-pandemic MAC samples. Not only were these values below the national average, but they were at the threshold value considered to be indicative of a high risk of depression, as defined by the Center for Epidemiological Studies Depression Scale (NHS, 2016; Taggart et al., 2015). The comparable baseline well-being scores may indicate that the health restrictions the public faced represented the lifestyle circumstances already being experienced by UK WIS personnel e.g., not attending mass gatherings and only going out when essential. Rarely leaving the house and avoiding social interaction were behaviors many of these participants were already familiar with as reported regarding their pre-course apprehensions: *“my anxiety means I haven’t been away in about 250 days,” “first time in 10 months I’ve been in a military group,” “being around other people again.”*

Importantly, an average increase in WEMWBS scores, reported by 261 participants pre-post R-MAC, was 13.52, suggesting an immediate program impact, even allowing for the COVID-19 adaptations. This improvement of 33% was comparable to the average increase achieved in the sample of 261 previous MAC attendees which was 36%. The increase reported by the 37 participants between pre-course values and six months later was 5.59. These improvements in mental well-being scores were not only statistically significant but also likely to represent real world “meaningful change” for the individuals. Although it is difficult to be sure about the level of change that is “meaningful,” best estimates range from three to eight WEMWBS scores for paired data sets (Putz et al., 2012). The sustained meaningful improvements reported at six-months are a testament to the deliberate attention on “transfer effects” within the R-MAC on participants’ mental well-being.

### Mitigating additional adverse effects of the COVID-19 pandemic for WIS personnel

Re-designing the MAC and developing the R-MAC may have helped to mitigate against further adverse effects of the COVID-19 pandemic and the imposed restrictions for the WIS participants. Primary evidence to support this consideration came out in written comments provided by R-MAC participants at the end of the course and during the follow-up study when asked if there was anything else they would like to add. These comments are a clear indicator of improved personal circumstances that were already being adversely affected by the pandemic, limiting the impact of which was critical for many (Holmes et al., 2020; Reger & Rothbaum, 2020).

### Participant resilience

Several studies have investigated the relationship between mental well-being and resilience. In survivors of traumatic abuse, a strong positive correlation and convergent validity was reported between WEMWBS and the Resilience Research Center-Adult Resilience Measure, which is indicative of the relationship and overlap between well-being and resiliency (Liebenberg & Moore, 2018). In research conducted with General Practitioners in Northern Ireland, WEMWBS scores were positively associated with four psychological resources: resilience, optimism, self-efficacy and, most strongly, hope (Murray et al., 2017). Therefore, it is reasonable to surmise that these R-MAC WEMWBS-assessed improvements in well-being are also indicative of improved resilience.

Thematic analysis revealed participants’ increased confidence and awareness of how to operate through the social distancing required in R-MAC. Paradoxically, COVID-19-oriented distancing protections may have reduced participants’ anticipation of the overall “demand” of every social engagement, leading to increased confidence for each engagement. This, in turn, may have encouraged further social connection. The psychological safety of this sense of “belonging,” and of being able to legitimately withdraw when demands become too much, may have allowed participants to focus on their recovery rather than on COVID-19 health guidance and the uncertainty surrounding the pandemic. Albeit socially distanced, the new and improved human connections intentionally activated by R-MAC peer support and health coaching, may have fostered new neural connections, which in turn provide potential for personal growth (Allan et al., 2012). This proposes an important program consideration; to design and embed experiences into the R-MAC that challenge participant’s resilience, rather than focusing on individuals’ efforts to develop their resilience. The impressive WEMWBS results emerging from the R-MAC, contextualized by our new findings regarding “social distancing,” have also made us reconsider our expectations for the types and levels of social interaction within the MAC.

### Limitations of the research

While taking part in an R-MAC, participants rarely decline to take part in the research surveys. When they leave however, 34% respond to research requests via e-mail two weeks later and 23% respond six months after attending. In future studies more can be done to improve this response rate such as utilizing contact methods other than e-mail, such as SMS or written surveys. Equally, because the sample is based on self-referring individuals, the follow-up data may represent a distinctively responsive group. Additionally, while no incentives were used to increase participation, this could be trialed in future studies. Ethical considerations would need to be made around undue inducement, exploitation, and biased enrollment.

The pre-course baseline measures of mental well-being aim to understand the pre-course engagement circumstances of the participants. Although they have not yet taken part in any of the course, they have travelled and enrolled, which could affect pre-course anxieties. Future studies could consider the use of wait-list control measures, by which participants who are due to attend a course provide measures of well-being in the lead-up to attending the course rather than on arrival, thus becoming their own control group.

### Resilience and readiness of the R-MAC service providers

Following its initial closure, the Battle Back Center remained open for the entirety of the pandemic, beyond one short closure due to government restrictions on travel in October 2020. Delivery of courses was unaffected by staff shortages, which confirms adherence to COVID-19 health guidance. The formal workflow matrix, developed by stakeholders to aid staff decision making, allowed for the rapid interpretation of current health guidance to immediately influence the delivery of the R-MAC.

The workflow matrix allowed for continuous reviews of delivery, underpinned by the varying levels of health guidance, as outlined by the government, and advised by Public Health England, NHS, sporting NGBs, MoD, SERCO on behalf of Sport England. With ongoing changes in health guidance, R-MAC providers were quickly able to adjust delivery. Although the Battle Back Center closed at the onset of the pandemic, the PRC delivered eight-, 12-, and 16-participant courses over four- and five-days. Importantly, the first day of every R-MAC included time dedicated to updating and familiarizing staff on current requirements for safe delivery.

In terms of future readiness, offering a reduced capacity and socially- distanced, yet socially meaningful, course will provide a flexible delivery option that still yields impactful behavior and well-being changes (Vindegaard & Benros, 2020).

## Conclusion

This research addressed the critical need to mitigate adverse health impacts arrising from COVID-19 for recovering UK military personnel, by evidencing an impactful recovery support course. The R-MAC led to sustained statistically significant improvements and meaningful change in participants' well-being and recovery, up to six-months after course completion. New evidence has been identified regarding the mechanisms of the intervention attributable to the positive changes in mental well-being. Precautions implemented to safeguard against COVID-19 transmission were satisfactory amongst participants and did not impede the recovery support. The development and success of the R-MAC, with service providers ability to adapt to continuous changes in delivery needs, has greatly improved the readiness and resilience to maintain vital support for recovering military personnel in the face of future challenging circumstances.

## Data Availability

Data available on request from the authors.

## Note

1. Taken from the Defense Recovery Capability Review 2020. This document is restricted and deemed “Official-Sensitive”. Although this is review document is not publicly available in its entirety, the author has granted permission for this comment to be quoted.

## Appendix 1

Open-ended questions included in the follow-up research surveys:

*Q. What part of the Reduced Numbers Multi Activity Course had the greatest impact on you?*

*Q. Please use this space to write any additional comments.*
